# Comments on John D. Keen and James E. Keen, What is the point: will screening mammography save my life? *BMC Medical Informatics and Decision Making*, 2009

**DOI:** 10.1186/1472-6947-9-20

**Published:** 2009-04-02

**Authors:** Michael Retsky

**Affiliations:** 1 Harvard Medical School, 300 Longwood Avenue, Boston, MA 02115, USA

## Abstract

This paper by John D. Keen and James E. Keen addresses a thorny subject. The numerical findings and commentaries in their paper will be disturbing to some readers and seem to defy logic and well established viewpoints. It may well generate angry letters to the editor. However such numerical analysis and reporting including civil discussion should be welcomed and are the basis for informed decision making – something that is highly needed in this field.

## 

Keen and Keen find that, depending on the particular population subset, of the order of a thousand persons need to be screened for early detection of breast cancer in order to save one additional life [1]. The remainder who do not benefit from early detection can have side effects of which they name anxiety, loss of time, unnecessary procedures, and radiation. The numbers are somewhat worse for younger women. They also comment and imply that early detection of cancer is a business with a large market and we should keep that in mind when we read papers and when women are urged to have mammographic screening to improve the mortality and morbidity from breast cancer. As Keen and Keen note, benefits are often exaggerated and side effects are often minimized.

I have studied this field for a number of years from a far distant vantage point. My opinions in this subject are mostly in agreement with Keen and Keen. That is, their comments and numerical results are consistent with my knowledge and experience. However, the situation is far more scientifically complex and politically charged than as they describe. I hope readers will become interested in this fascinating subject and study the literature. Early detection of breast cancer is scientifically most interesting but unfortunately this is often lost in the arguing that has taken place.

Consider that we had a cancer paradigm consisting of detection of cancer by no special means and subsequent treatment by surgery and possible adjuvant therapy. Now we perturb or probe the system by introducing routine early detection. Do the new results on outcome agree with our expectations? If they do, then we gain confidence that we understand the disease and how it progresses. If they do not, then perhaps our understanding of the disease process was faulty and needs to be reconsidered. Such experimental opportunities are rare in cancer care so we need to fully exploit the scientific situation.

It has long been known that the probability of cure with just surgical removal of the primary tumor after diagnosis of breast cancer is highest for patients with the smallest primary tumors and no invasion of the axillary lymph nodes. The probability of cure decreases as the tumor size and the number of lymph nodes with cancer cells increases. It was eminently logical that early detection would be a benefit. Trials to determine the quantitative value of detecting cancer early began in the 1960s and still continue.

When they evaluated benefit of early detection of cancer for women age 50–59, there was an early appearing and strong 20 – 30% mortality advantage to early detection. This was expected. However, when they did the same thing for women age 40–49, there was an unexpected early mortality disadvantage to mammography that later became the anticipated advantage after 6 or more years. Essentially equivalent results were reported from a number of trials that occurred in different countries and over several decades of time.

This was not what people wanted to hear. When disputed and clinically important results occur, NIH often holds a consensus conference where all data are presented and an expert panel reviews data and makes recommendations. The majority of scientists who examined the trial data concluded there is no evidence supporting the use of mammography for women age 40–49. A vocal minority disagreed.

Since the trials of early detection of breast cancer encompass the entire cancer process from recruitment of supposedly healthy individuals, ensuing treatment, to long term survival or death presumably due to cancer, there are many opportunities for biases or potential biases to appear. It was easy to criticize the trials for actual or possible biases. By discarding these data, they were then free to make decisions based on *their *biases. Screening advocates concluded that routine mammography should be started at age 40 since, despite trial data, they were sure early detection is beneficial. The Director of the NIH as well as the US Senate (by a vote of 98 to 0) decided that the trials were wrong and mammography should start at age 40. For a colorful report of that NIH consensus conference, see the New England Journal of Medicine paper by Suzanne Fletcher in which the consensus conference is compared to Alice in Wonderland [2].

A number of years ago, based on a study of bimodal relapse patterns in clinical data, my colleagues and I proposed that breast cancer growth includes periods of quasi-stable dormancy and that surgery to remove the primary tumor can kick-start growth of dormant distant single malignant cells and micrometastases. According to our report, this is very common in that over half of all relapses in breast cancer are accelerated by such mechanisms. For premenopausal women with positive lymph nodes, there is a particularly high incidence of surgery-induced angiogenesis that results in very early relapses. These effects would reduce the benefit of early detection in the short term but not affect the long term benefit. For premenopausal women there would be a particularly sharp early effect. We calculated that based on clinical data, 0.1/1000 young women screened would relapse and die 2–3 years after the start of screening. This quantitatively explained the excess mortality for trials to test the benefit of early detection of breast cancer for women age 40–49. The theory also at least partly explained why there is only modest benefit of early detection of breast cancer in general [3].

In the trials, some apparently healthy young women died from breast cancer 3 years after mammography began. As frequently happens in this subject, the numbers are very small despite many women screened. According to a meta-analysis, there might barely be a statistically significant excess mortality at 3 years for young women. The disturbing implication is that most people derive benefit from early detection however other people especially young women may die earlier as a result of early detection. It must be emphasized that modern adjuvant therapy moderates these early events to some degree.

There is also the possibility that the extra biopsies resulting from extra imaging studies could produce extra patients with more positive lymph nodes as another possible side effect of routine mammography [4].

Our papers on this subject generated several news reports. In one major web report, the director of screening for American Cancer Society (ACS) is quoted as saying readers should not believe any of this [5,6]. ACS provides a very valuable service to the community but we might keep in mind that this is a multi-billion dollar market and allegedly there are historic ties to the mammography and radiology communities [7].

One of our papers on early detection generated an angry and insulting letter to the editor [8] by a noted mammography advocate who has written similar letters before [9]. Such an environment is not helpful for unbiased scientific research.

Of course there are many knowledgeable and well-meaning advocates who will disagree with the findings of Keen and Keen. However, as one recent very negative report presenting data along the lines of the Keen and Keen findings, Gotzsche et al [10] note that "If 2000 women are screened regularly for 10 years, one (additional person) will benefit from the screening, as she will avoid dying from breast cancer. At the same time, 10 healthy women will, as a consequence, become 'cancer patients' and will be treated unnecessarily."

We have all been told many times that mammography saves lives. That is certainly true but it also may result in other things that are not beneficial and we need a decision algorithm that includes more of these effects. The study by Keen and Keen is a positive step in the right direction. As Dr. Cornelia Baines (co- PI of a large trial of early detection for women age 40–49) writes, women age 40–49 are asked to sign informed consent for mammography without being properly informed [11].

## Competing interests

I occasionally consult in lawsuits involving delayed diagnosis of cancer. I also have submitted a patent application for a therapy for early stage breast cancer that does not produce surgery-induced metastatic growth [12]. I am a founder and on the Board of Directors of the Colon Cancer Alliance [13] for which I receive no compensation. Early detection of colon cancer has its own set of problems but it seems that dormancy and surgery-induced metastatic growth are at most small effects in early stage colon cancer compared to breast cancer. I think there should be more effort to increase early detection of colon cancer for men and women over age 50 and full information should be provided to women regarding possible serious side effects of early detection of breast cancer especially for women under age 50.

## Pre-publication history

The pre-publication history for this paper can be accessed here:

http://www.biomedcentral.com/1472-6947/9/20/prepub
